# Lack of Phenotypic and Evolutionary Cross-Resistance against Parasitoids and Pathogens in *Drosophila melanogaster*

**DOI:** 10.1371/journal.pone.0053002

**Published:** 2012-12-21

**Authors:** Alex R. Kraaijeveld, Sophie J. Layen, Peter H. Futerman, H. Charles J. Godfray

**Affiliations:** NERC Centre for Population Biology, Imperial College London, Silwood Park Campus, London, United Kingdom; University of Cambridge, United Kingdom

## Abstract

**Background:**

When organisms are attacked by multiple natural enemies, the evolution of a resistance mechanism to one natural enemy will be influenced by the degree of cross-resistance to another natural enemy. Cross-resistance can be positive, when a resistance mechanism against one natural enemy also offers resistance to another; or negative, in the form of a trade-off, when an increase in resistance against one natural enemy results in a decrease in resistance against another. Using *Drosophila melanogaster*, an important model system for the evolution of invertebrate immunity, we test for the existence of cross-resistance against parasites and pathogens, at both a phenotypic and evolutionary level.

**Methods:**

We used a field strain of *D. melanogaster* to test whether surviving parasitism by the parasitoid *Asobara tabida* has an effect on the resistance against *Beauveria bassiana*, an entomopathogenic fungus; and whether infection with the microsporidian *Tubulinosema kingi* has an effect on the resistance against *A. tabida*. We used lines selected for increased resistance to *A. tabida* to test whether increased parasitoid resistance has an effect on resistance against *B. bassiana* and *T. kingi*. We used lines selected for increased tolerance against *B. bassiana* to test whether increased fungal resistance has an effect on resistance against *A. tabida*.

**Results/Conclusions:**

We found no positive cross-resistance or trade-offs in the resistance to parasites and pathogens. This is an important finding, given the use of *D. melanogaster* as a model system for the evolution of invertebrate immunity. The lack of any cross-resistance to parasites and pathogens, at both the phenotypic and the evolutionary level, suggests that evolution of resistance against one class of natural enemies is largely independent of evolution of resistance against the other.

## Introduction

Virtually all organisms suffer from attack by natural enemies, and the vast majority will face more than one species of natural enemy, be it predators, herbivores, parasites and/or pathogens. As natural enemies reduce the fitness of their victim, it is expected that victims are under selection to evolve a mechanism allowing them to defend against attack by their natural enemies, or avoid attack altogether.

When organisms face attack by different types of natural enemies, e.g. by predators and parasites, the defense mechanism evolved as a response to selection by one natural enemy may have an effect on defense against a second one. Such cross-resistance can be positive, when resistance evolved in response to one natural enemy is effective against another one. An example of this would be running speed in a prey. If a prey evolves to run faster in response to one predator, this faster running speed will usually make it less likely to be caught by another predator. In plants there is evidence for cross-resistance to pathogens and herbivores [1], possibly due to the cross-talk amongst key signalling pathways [2]. Mice infected with *Schistosoma mansoni* showed no change in resistance when subsequently infected with *Schistosomatium douthitti*, whereas mice infected first with *Schistosomatium douthtti* were more resistant to *Schistosoma mansoni* compared to the controls [3].

Cross-resistance can also be negative and take the form of a trade-off, when increased resistance against one type of natural enemy results in a decrease in resistance against another type of natural enemy. For instance, in the snail *Lymnaea stagnatilis*, stimulation of predator-avoidance behaviour (expelling of blood followed by retreat into the shell) resulted in a reduction in the proportion of phagocytotic haemocytes, which are important in defense against pathogens [4]. Although not explicitly tested, the underlying assumption is that this results in a reduced ability to defend against pathogens.

Cross-resistance can occur at a phenotypic level and at an evolutionary level. We will focus on immunological defense to explain this further. At the phenotypic level, we consider the *actual* launching of an immune response; at the evolutionary level, we consider the *ability* to launch an immune response as a result of investment in the immunological machinery. Cross-resistance at the phenotypic level implies that activation of the immune system due to infection by a parasite or pathogen has an effect on the subsequent immune response against infection by a second parasite or pathogen. A positive effect can be due to priming, when the immune system is upregulated by the first infection, resulting in an increased level of immunity against the second infection. Despite the fact that invertebrates do not possess an adaptive immune system in the way that vertebrates do, priming has been found in invertebrates, even across generations [5]–[7]. A negative effect would indicate either a pleiotropic effect, when expression of genes involved in an immune reaction against one parasite or pathogen has a negative effect on the immune reaction against another parasite or pathogen; or allocation costs, when resources already used up by the immune response against the first infection cannot be used again in the immune reaction against the second infection.

Cross-resistance at the evolutionary level is found when the ability to launch an immune response against one type of parasite or pathogen is linked to the ability to launch an immune response against another type of parasite or pathogen. If this link is positive, it suggests that the immunological pathways against the two types of parasite or pathogen are identical, or at the very least shared to a substantial degree. A negative link suggests the existence of a trade-off in the host's immune system. An increased ability to launch an immune response against one type of parasite or pathogen then leads to a decreased ability to launch an immune response against another type. For instance, humans carrying an allele of the DARC gene which makes them more resistant to malaria are more susceptible to HIV [8]. Again, such a trade-off could be the result of pleiotropic or allocation effects.

*Drosophila melanogaster* and its parasites and pathogens are a very important and useful model system for understanding the ecology and evolution of invertebrate immunity (see [9]–[10] for recent reviews). Larvae are parasitized by hymenopteran parasitoids, of which the braconid *Asobara tabida* and the figitids of the genus *Leptopilina* are most common in Europe [11]–[12]. The immune response launched by *D. melanogaster* larvae in response to parasitoid attack has both a cellular and a humoral aspect [13]–[16]. After the parasitoid egg is oviposited into the host, it is enveloped by the host's haemocytes, especially by lamellocytes. Subsequently, another type of haemocytes, crystal cells, release enzymes which trigger the prophenoloxidase cascade. The end result of this cascade is the deposition of melanin around the parasitoid egg, which dies if the capsule is completely closed [17]. Parasitoids have different mechanisms to overcome this immune response. *A. tabida* has eggs with a ‘sticky’ egg chorion, which causes the eggs to become hidden in host tissue, away from circulating haemocytes [17], [18]. Parasitoids of the genus *Leptopilina* inject virus-like particles (VLPs) into the host, which either block production of haemocytes, or cause their alteration or apoptosis [19]–[22].

In the field, there is considerable variation in the ability of *D. melanogaster* to encapsulate parasitoid eggs [23]. However, resistance against *A. tabida* and *L. boulardi* was not correlated across field strains [23]. In contrast, there are positive correlations across isofemale lines in resistance against *L. boulardi* and *L. heterotoma* [24], [25]. Using lines selected for resistance against *A. tabida* and *L. boulardi*, cross-resistance was found to be asymmetrical [26]: selection for resistance against *A. tabida* did not lead to increased resistance against *L. boulardi*, whereas selection for resistance against *L. boulardi* resulted in increased resistance against *A. tabida*. Both sets of selection lines were more resistant against *L. heterotoma* than their respective controls.

Pupal parasitoids of *D. melanogaster* avoid the host's immune system by laying their eggs outside of the actual pupa, though inside the puparium. Puparium cuticle formation and parasitoid egg encapsulation are two processes that share resources and pathways [27], [28], opening up the possibility of cross-resistance against larval and pupal parasitoids. Females of the common pupal parasitoid *Pachycrepoideus vindemiae* preferentially attacked pupae which had previously parasitized by *A. tabida* [29], presumably due to a thinner puparial wall; this effect was found in young pupae, though not in older pupae. However, selection for resistance to a larval parasitoid (*A. tabida*) was found to have no effect on the probability of parasitism by *P. vindemiae* [30]. Similarly, no correlation was found across isofemale lines for resistance against larval and pupal parasitoids [25].

Adults of *D. melanogaster* are susceptible to infection by entomopathogenic fungi, such as *Beauveria bassiana* [31], [32]. In order to defend itself against microbial pathogens, *Drosophila* employs a battery of antimicrobial peptides [33]–[36], even though haemocytes also play a role [37], [38]. Exposing replicated populations of *D. melanogaster* to *B. bassiana* resulted in flies evolving tolerance rather than actual resistance: after infection, flies from the selected lines did not live longer than flies from the unselected control lines, but maintained their fecundity for longer [32].

The microsporidian *Tubulinosema kingi* can infect *D. melanogaster* as well as its parasitoids [39]. Flies, when exposed to the *T. kingi*, evolve a degree of resistance against this pathogen [40]: selection lines suffer less of a reduction in fecundity than control lines. However, nothing is known about the resistance mechanism that *D. melanogaster* employs against microsporidia. In other insect species, both haemocytes and the prophenoloxidase/melanization system have been implicated [41]–[44].

As detailed above, some work has been done on *D. melanogaster* cross-resistance against different parasitoid species. However, nothing is known about the link between resistance to parasitoids and microbial pathogens, which use largely different arms of the immune system (haemocytes vs anti-microbial peptides, respectively). In this paper, we aim to investigate cross-resistance to parasitoids and pathogens in *D. melanogaster*, both at the phenotypic and the evolutionary level. At the phenotypic level, we will test whether surviving parasitism by the parasitoid *A. tabida* as larvae affects adult resistance to the fungus *B. bassiana*, and whether larval infection with the microsporidium *T. kingi* affects subsequent larval encapsulation of *A. tabida* eggs. This latter case could also be regarded as co-infection, even though infection with the microsporidium occurs before parasitism, as it is unknown how fast any immune response against microsporidia is launched by the larvae. At the evolutionary level, we will test whether selection for larval resistance to *A. tabida* affects adult resistance to *B. bassiana* and to *T. kingi*, and whether selection for adult tolerance to *B. bassiana* affects larval resistance to *A. tabida*.

## Materials and Methods

### Fly, parasitoid, fungus, and microsporidian cultures

We used *D. melanogaster* strain AV for the phenotypic level experiments; this strain was originally collected in Avigliano, Italy [45]. For the evolutionary level experiments, we used two sets of paired control and selection lines. One set consisted of four lines selected for increased resistance against *A. tabida* and their controls [46]. A few months before the experiments described in this paper, the encapsulation ability of these lines, and their paired control lines, was re-measured; this confirmed that the selection lines had a higher rate of encapsulation than the control lines (39% vs 18%, respectively). The second set consisted of five lines selected for increased tolerance to *B. bassiana* [32] and their controls. These flies were used in the experiments described here within a few months of completion of the selection procedure. All flies were reared in 150 ml bottles with yeast/sugar medium and live baker's yeast (see [12] for more details).

We used the SOS strain of *A. tabida*, which was originally collected near Sospel, southern France and has been cultured in the laboratory for many years on *D. subobscura* (see [12] for more details).

*B. bassiana* (strain 80.2) was stored at −80°C in spore suspension form (10^7^ spores/ml in 25% glycerol) and cultured on Saboraud Dextrose Agar (SDA) plates (see [32] for more details).

*T. kingi* was not cultured in vivo, but extracted from heavily-infected individuals in the *A. tabida* culture [39]. When microsporidia were needed for an experiment, we extracted spores from the parasitoids by homogenising them in 0.1% SDS and filtering the homogenate to remove parasitoid tissue (see [39] for more details).

All flies and parasitoids were cultured at 20±1°C, the fungus at 29±0.5°C.

### Resistance against fungi of flies surviving parasitism

We let flies lay eggs and when the larvae had reached the 2^nd^ instar, we transferred a few hundred larvae to each of 30 fresh bottles. We added five female parasitoids to 20 of the bottles for one day. When the larvae had pupated, we checked those which had been exposed to parasitoids under a microscope for signs of encapsulated parasitoid eggs (which are clearly visible through the puparial wall), indicating they had been parasitised and had survived this. We collected 2000 such pupae, plus a further 1000 random pupae from the ten bottles which were not exposed to parasitoids. We kept pupae in groups of 20 in vials with medium and yeast at 20±1°C. Two to four days after fly emergence, we created six batches of capsule-carrying flies and six batches of unexposed flies; each batch contained about 100 flies. Flies in three of the capsule-carrying and three of the unexposed batches were infected with fungus by gently shaking them for 10–15 seconds in a plate with a sporulating fungus colony [32]. To allow us to use the same fungus plate for the capsule-carrying and control flies, we divided the plate into two arenas separated by a strip of acetate sheet. Flies from the remaining three encapsulated and three unexposed batches were gently shaken in a similarly divided Petri-dish lined with a piece of filter paper and served as treatment controls. After shaking, all flies were released into Perspex cages (25×25×25 cm) with a muslin sleeve at the front and containing honey and water at all times (one cage was used for every batch of flies). Cages were kept at 20±1°C and checked every day; dead flies were removed and counted. After 40 days we ended the experiment and counted any still living flies. The experiment was done in two blocks, comprising a total of 12 batches of capsule-carrying flies and 12 batches of unexposed flies.

For each cage we fitted a Weibull distribution through the survival data, including flies still alive at the end of the experiment as censored. From the scale (α) and shape (γ) parameters, we calculated time to death (TTD) in each of the cages as TTD  =  α Γ (1/γ +1) [47]. We analysed times to death in a 3-way ANOVA with block, encapsulation status and fungal infection as factors.

### Encapsulation ability of larvae infected with microsporidia

A microsporidia-free line was created as described in detail in [39]. Once this line was established, flies were given bottles with medium and yeast to lay eggs. To half the bottles we added a suspension of microsporidian spores in 0.1% SDS and to the other half only 0.1% SDS. When the larvae had reached the 2^nd^ instar, we added 20 infected larvae to each of 10 small Petri-dishes lined with agar and containing a patch of a thin yeast suspension, and 20 uninfected larvae to each of 10 further Petri-dishes. Then we introduced two parasitoid females (less than 2 weeks old) into each of the dishes for two hours. Dishes were incubated at 20±1°C.

Five days later we dissected all larvae and pupae and scored encapsulated and non-encapsulated parasitoid eggs and larvae. We calculated encapsulation ability as the proportion of singly-parasitised larvae that had successfully encapsulated the parasitoid egg [23]. We repeated this experiment five times, giving five pairs of encapsulation abilities (from infected and uninfected larvae), based on a total of 657 singly-parasitised larvae. We compared encapsulation abilities of infected and uninfected larvae using GLM, specifying a binomial error structure, and with ‘pair’ fitted first to take the paired experimental set-up into account.

### Resistance against fungi of flies selected for parasitoid resistance

For logistical reasons, this experiment was performed differently from the one described above. Fifty flies (2–4 days old) from each of the four pairs of selection and control lines were gently shaken in a Petri-dish with a sporulating fungus colony for 10–15 seconds; 50 additional flies from each line were shaken in a Petri-dish with filter paper. After shaking, we transferred the flies to vials with medium and live yeast (10 flies per vial) and kept them at 20°±0.5C. We scored dead flies daily and replaced the vials every 2 days until all flies had died.

We fitted Weibull curves through the data from each vial and estimated time to death (TTD) for each vial as described above. Then we analysed average times to death for each of the control and selection lines with a 2-way ANOVA, with selection history and infection treatment as factors.

### Resistance to microsporidia of flies selected for parasitoid resistance

We first treated all four pairs of selection and control lines as described in [39] to make sure that lines were free of microsporidian infection prior to the experiment. We then let flies from each of the eight infection-free lines lay eggs. For each line, we placed 60 eggs in each of 10 vials containing medium and live yeast. The following day, we added a spore suspension in 0.1% SDS to half of the vials as described above; the remaining vials only received 0.1% SDS and acted as controls.

After flies had emerged, we allowed them to mate and mature eggs for five days. Then we randomly picked two females from each vial and placed those individually in fresh vials with medium and live yeast. To each of these vials we added two males. We transferred each trio of flies to a fresh vial daily and counted numbers of eggs in the vials from the previous day for a period of 10 days (fecundity is a proxy measure for resistance against microsporidia [39]). Dead males were removed and replaced. We then calculated mean daily fecundity for each female to give us average early fecundity for each of the selection and control lines. These were analysed with a 2-way ANOVA with selection history and infection treatment as factors.

### Encapsulation ability of larvae selected for fungal tolerance

For each of the five pairs of lines, we added 20 2^nd^ instar selection larvae to each of 10 small Petri-dishes lined with agar and containing a patch of a thin yeast suspension and 20 control larvae to each of 10 further Petri-dishes. Introduction of parasitoid females, incubation, dissection and analysis was exactly as described above (the experiment gave us a total of 642 singly-parasitised larvae).

## Results

### Fungal resistance of flies surviving parasitism

**Figure 1:**
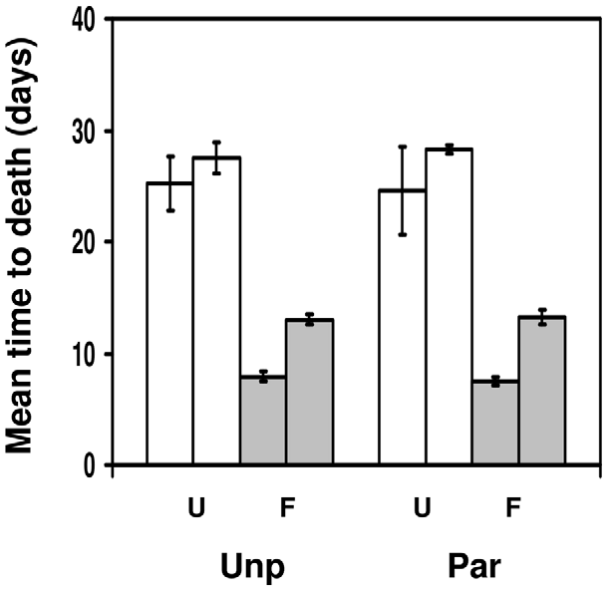
Fungal resistance of flies surviving parasitism. Mean time to death (in days) for *Drosophila melanogaster* adult flies that were either infected with *Beauveria bassiana* (F; grey bars) or uninfected (U; white bars), depending on whether they had been parasitised by *Asobara tabida* as a larvae (Par; right side of panel) or had not been parasitised (Unp; left side of panel). The two bars within each treatment combination represent the results of the two experimental blocks. Bars show mean ± s.e.

This experiment was set up to test whether survivorship of adult flies after fungal infection is affected by whether these flies had suffered and survived parasitism as larvae. Fig. 1 shows the effect of fungal infection on survivorship of flies which have successfully encapsulated a parasitoid egg or have not been attacked by *A. tabida*. Fungus infection had a strong and very significant effect on survivorship (F_1,16_ = 167.047, p<<10^−6^). There was no significant effect of parasitism status (F_1,16_ = 0.0006, p = 0.981), but there was a significant block effect (F_1,16_ = 11.737, p = 0.0035), with flies in the second block surviving longer. The most important statistic is the interaction between parasitism status and infection treatment. This interaction was not significant (F_1,16_ = 0.0034, p = 0.954), which shows that previous attack by parasitoids and subsequent larval encapsulation of the parasitoid egg had no effect on how long adult flies survive after fungal infection. None of the other interactions were significant (the 3-way parasitism status x infection status x block interaction gave F_1,16_ = 0.03, p = 0.86). Focusing on infected flies only, unparasitised flies lived 0.4 days longer than parasitised flies in block 1, wheres parasitised flies lived 0.2 days longer than unparasitised flies in block 2. In both cases, the 95% confidence intervals around the difference in time to death included zero (−1.2 to 2.0 days in block 1; −2.0 to 2.4 days in block 2).

### Encapsulation ability of larvae infected with microsporidia

**Figure 2:**
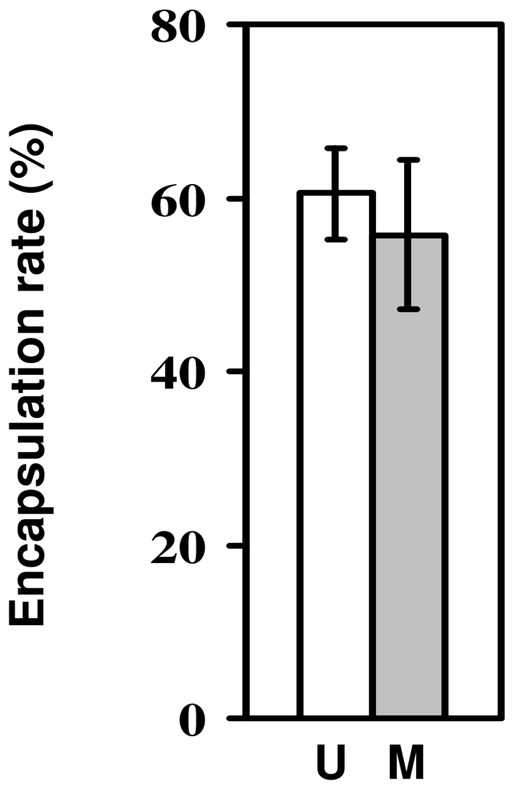
Encapsulation ability of larvae infected with microsporidia. Rate of encapsulation of *Asobara tabida* eggs by *Drosophila melanogaster* larvae which had either been infected with *Tubulinosema kingi* (M; grey bar) or had not been infected (U; white bar). Bars show mean ± s.e.

We set up this experiment to test whether infection with microsporidia affected the rate of encapsulation of parasitoid eggs. Overall, the larvae encapsulated 55–60% of the parasitoid eggs (fig. 2). Although the encapsulation rate of larvae infected with microsporidia was slightly lower than that of uninfected larvae, this difference was far from significant (change in deviance  = 0.38, d.f = 1, p = 0.54), showing that microsporidian infection had no effect on larval encapsulation of parasitoid eggs. Infected flies had a 4.8% higher encapsulation rate than uninfected flies, but the 95% confidence intervals around this difference in encapsulation ability (−18.3% to 27.8%) included zero.

### Fungal resistance of flies selected for parasitoid resistance

**Figure 3:**
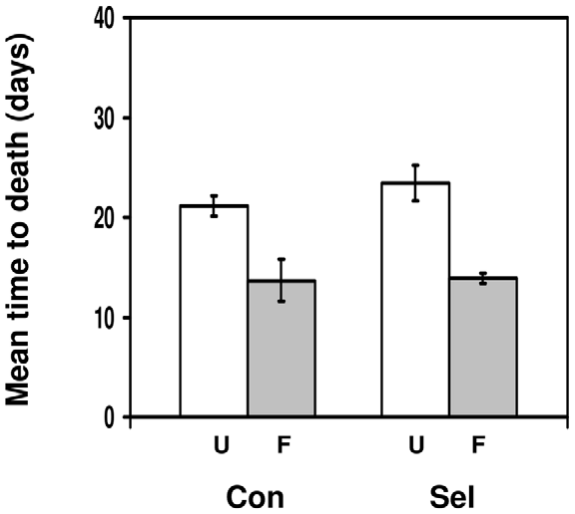
Fungal resistance of flies selected for parasitoid resistance. Mean time to death (in days) for *Drosophila melanogaster* adult flies that were either infected with *Beauveria bassiana* (F; grey bars) or uninfected (U; white bars), depending on whether they had been selected for increased resistance to *Asobara tabida* (Sel; right side of panel) or had not been selected (Con; left side of panel). Bars show mean ± s.e.

In this experiment, we tested whether the ability to encapsulate parasitoid eggs as larvae influences adult survival after fungal infection. As in the earlier fungal experiment, the time to death of flies was much reduced after fungal infection (fig. 3; F_1,12_ = 31.47, p = 0.00011). There was no significant difference in time to death between control and selection flies (F_1,12_ = 0.672, p = 0.43) and no significant interaction between selection history and infection treatment (F_1,12_ = 0.496, p = 0.49). This shows that having a higher level of larval resistance to parasitoids had no effect on adult survival after fungal infection. Focusing on infected flies only, selected flies lived 0.2 days longer than control flies, but the 95% confidence intervals around the difference in time to death (−5.3 to 5.6 days) included zero.

### Resistance to microsporidia of flies selected for parasitoid resistance

**Figure 4:**
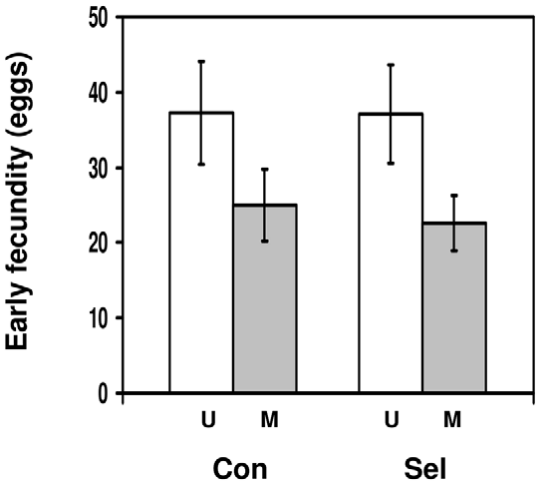
Resistance to microsporidia of flies selected for parasitoid resistance. Early fecundity of *Drosophila melanogaster* females adult flies that were either infected with *Tubulinosema kingi* (M; grey bars) or uninfected (U; white bars), depending on whether they had been selected for increased resistance to *Asobara tabida* (Sel; right side of panel) or had not been selected (Con; left side of panel). Bars show mean ± s.e.

This experiment was set up to test whether the ability to encapsulate parasitoid eggs as larvae affects resistance against microsporidia, using fecundity as a proxy for resistance. Fly fecundity was reduced by microsporidian infection (Fig. 4; F_1,12_ = 5.62, p = 0.035), but there was no difference between control and selection flies in their fecundity (F_1,12_ = 0.053, p = 0.822) and no significant interaction between selection history and infection treatment (F_1,12_ = 0.041, p = 0.84). In other words, flies selected for increased parasitoid resistance and unselected control flies suffered the same reduction in fecundity after microsporidian infection. Focusing on infected flies only, control flies laid 2.5 eggs more than selected flies, but the 95% confidence intervals around the difference in fecundity (−12.4 to 17.3 eggs) included zero.

### Encapsulation ability of larvae selected for fungal tolerance

**Figure 5:**
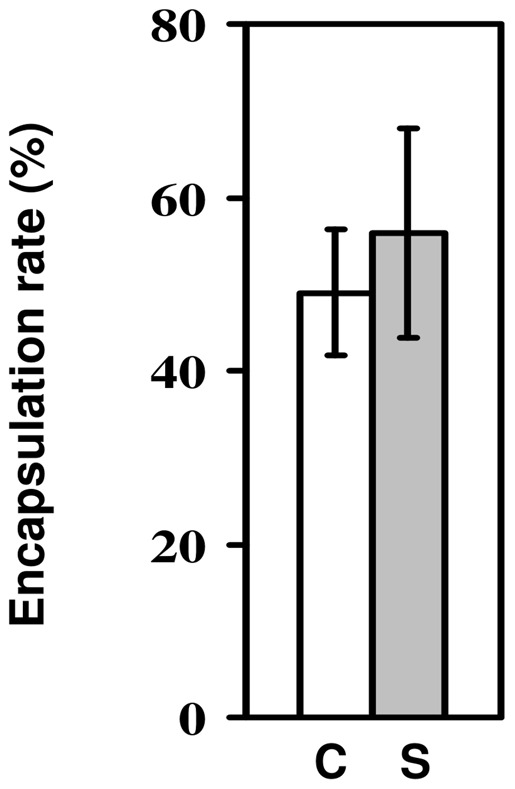
Encapsulation ability of larvae selected for fungal resistance. Rate of encapsulation of *Asobara tabida* eggs by *Drosophila melanogaster* larvae which had either been selected for tolerance to *Beauveria bassiana* (S; grey bar) or had not been selected (U; white bar). Bars show mean ± s.e.

We set up this experiment to test whether selection for adult fungal tolerance has an effect on the ability of larvae to encapsulate parasitoid eggs. Larvae from lines selected for fungal tolerance and control lines encapsulated 50–55% of the eggs (fig. 5). Although the encapsulation rate of larvae from the selected lines was slightly higher than that of larvae from the control lines, this difference was far from significant (change in deviance  = 2.11, d.f. = 1, p = 0.14), showing that selection for adult fungal tolerance had no effect on larval encapsulation of parasitoid eggs. Selected flies had a 6.9% higher encapsulation rate than control flies, but the 95% confidence intervals around the difference in encapsulation ability (−25.7% to 39.5%) included zero.

## Discussion

We set out to explore whether *D. melanogaster* shows cross-resistance to parasites and pathogens. We explored cross-resistance at the phenotypic level, when the *actual* immune response after infection by one parasite or pathogen has an effect on the immune response after infection by a subsequent parasite or pathogen, and at the evolutionary level where the *ability* to activate an immune response against one parasite or pathogen has an effect on the immune response against another parasite or pathogen. At a phenotypic level, we found that successful larval encapsulation of parasitoid eggs has no effect on adult resistance to fungal infection, and larval infection with microsporidia has no effect on subsequent encapsulation of parasitoid eggs. Depending on the speed at which the larvae launch an immune response against microsporidia, this could also be a case of co-infection. However, the finding remains that encapsulation of parasitoid eggs is not affected by infection with microsporidia. At an evolutionary level, we found that selection for parasitoid resistance has no effect on resistance to microsporidia or fungi, and selection for tolerance to fungi has no effect on effect on resistance to parasitoids. We conservatively considered the level of replication to be at the line, cage, or dish level rather than at the level of the individual larva or fly, in order to avoid any pseudoreplication. Even though our analyses have relatively low power as a result of this conservative approach to the level of replication, all relevant main and/or interaction effects were far from significance. Despite all our results being negative, we feel that they are important, given the use of *D. melanogaster* as a key model system for the evolution of invertebrate immunity. Also, to the best of our knowledge, this is the only data-set on a single host species showing a consistent lack of a link between resistance to parasites and pathogens. This absence occurs both at the phenotypic and at the evolutionary level.

To enable an easy comparison between our findings and earlier findings on cross-resistance in *D. melanogaster*, we combined all findings in table 1. The ‘positive/none’ entry for cross-resistance to larval parasitoids of different genera refers to the asymmetric cross-resistance of lines selected for resistance to *A. tabida* and *L. boulardi*, where *L. boulardi*-selected lines show increased resistance to *A. tabida*, but not vice versa [23]. The ‘negative/none’ entry for cross-resistance between larval and pupal parasitoids refers to the effect of age, where negative cross-resistance was found in young pupae, whereas no effect was found in older pupae [29].

**Table 1:** Cross-resistance in *Drosophila melanogaster*.

*Resistance against:*	*Phenotypic cross-resistance*	*Evolutionary cross-resistance*
Larval parasitoids of same genus		**positive**
Larval parasitoids of different genera		**positive**/**none**
Larval and pupal parasitoids	**negative**/**none**	**none**
Larval parasitoids and fungal pathogens	none*	none*
Larval parasitoids and microsporidian pathogens	none*	none*

Overview of the existence of cross-resistance, and its direction if present, in *Drosophila melanogaster* in response to various combinations of parasites and pathogens. Based on data from this paper (indicated by an *) and the literature (none  =  no cross-resistance detected; empty cell  =  cross-resistance not tested).

At a phenotypic level, mealworms (*Tenebrio molitor*) challenged with LPS had higher resistance to fungal infection [48], and bumblebees (*Bombus terrestris*) which were challenged with LPS (lipopolysaccharide, a bacterial antigen), showed higher levels of antibacterial activity but lower phenoloxidase activity [49]. As in the case of the snail *Lymnaea stagnatilis* [4], the assumption is that this lower level of phenoloxidase activity indeed leads to a reduced ability to defend against parasites and/or pathogens. Cross-resistance between parasitoids and fungal pathogens comes closest conceptually in our experiments with *D. melanogaster*, given that the host uses phenoloxidase and antimicrobial pathogens against these two challenges, respectively. We found no evidence of cross-resistance between parasitoids and fungal pathogens. However, the bumblebees were first challenged with LPS before their level of phenoloxidase activity was assessed. In our experiments with *D. melanogaster*, the two challenges were given the other way around, and to two different life stages (larvae and adults). In the gypsy moth (*Lymantria dispar*), the spore load of the microsporidium *Vairimorpha* sp. in infected larvae parasitised by the parasitoid *Glyptapanteles liparidis* is higher than in infected unparasitised larvae [50], [51]. This is in contrast with the lack of any cross-resistance against microsporidia and parasitoids in *D. melanogaster*, but again, the order of the challenges is reversed. In the case of such negative phenotypic cross-resistance, it is difficult to separate actual immune-based trade-offs from a general decrease in health. Microsporidian spore load being higher in gypsy moth larvae parasitized by a parasitoid could be interpreted as a case of the immune response against one parasite trading off with the immune response against the other. However, the same pattern might be caused by the polydnaviruses of the parasitoid suppressing the host's immune system, thereby allowing the microsporidium to proliferate faster, or alternatively by the larvae suffering from a general decline in condition as a result of being parasitised. More detailed work on exactly what happens at an immunological level is required to differentiate among these hypotheses.

At an evolutionary level, fall army worm (*Spodoptera frugiperda*) selected for resistance to *S. frugiperda* Nuclear Polyhedrosis Virus (NPV) were less susceptible to *S. frugiperda* Granulosis Virus (GV) and *Autographa californica* NPV [52]. Similarly, among pea aphid (*Acyrtosiphon pisum*) clones, resistance against the parasitoid *Aphidius ervi* was positively correlated with resistance against *Aph. eadyi* [53]. These results match the positive cross-resistance against different parasitoid species in *D. melanogaster*. Cross-resistance against the fungal pathogen *Pandora* (formerly *Erynia*) *neoaphidis* and either *A. ervi* or *A. eadyi* was borderline positive [53]. This result is not matched in *D. melanogaster*, where there is no evidence of cross-resistance between parasitoids and fungal pathogens.

Positive cross-resistance against two parasites and/or pathogens is most likely to occur when the immune response against both uses the same physiological and biochemical mechanisms whereas trade-offs are more likely to occur when different mechanisms are employed. In *Spodoptera littoralis*, a negative genetic correlation was found between antibacterial activity and haemocyte density, whereas there were positive genetic correlations between haemocyte density, phenoloxidase activity and cuticular melanisation [54]. In *D. melanogaster*, resistance against parasitoids largely involves a cellular response that uses the phenoloxidase pathway leading to melanisation of the parasitoid egg [13]–[17]. Resistance against fungi (and bacteria) is predominantly mediated by anti-microbial peptides [33]–[36], whereas we know nothing of the resistance mechanism employed against microsporidia. In addition, at a genetic level, there is only limited overlap between the genes involved in the immune response against parasitoids and that against microbes [45]. Given that resistance against parasitoids and against fungi use different mechanisms, we might not expect positive cross-resistance between parasitoids and fungi, and indeed we did not find any. On the other hand, positive cross-resistance was found against two larval parasitoid species, against which the same immune mechanism is involved [24]–[26]. However, trade-offs can still occur different immune mechanisms are involved, as both require energy and possibly other inputs. A modelling approach showed that attack by multiple enemies can lead both to positive and negative cross-resistance [55], depending on the nature of the specific defense mechanisms, the costs of these defense mechanisms, and the encounter rates with the enemies. Although a lot is known about defense mechanisms against a wide variety of natural enemies, very little knowledge exists about the costs of these defense mechanisms. Similarly, data on encounter rates with natural enemies in the field are scarce. In order to arrive at a more general and more predictive understanding of the cross-resistance of organisms against their natural enemies, more case studies are required first of all. More importantly, increased knowledge of immunological and other defense mechanisms and the costs associated with them is needed before we can predict under which circumstances we would predict to see positive, negative, or a lack of cross-resistance when different arms of the immune system are involved.
